# Plant resistance and predators influence the density dependence of herbivore survival and distribution of herbivory

**DOI:** 10.1002/ece3.70106

**Published:** 2024-08-07

**Authors:** Monica Paniagua Montoya, Alexander J. Forde, Brian Inouye, Nora Underwood

**Affiliations:** ^1^ Department of Biological Science Florida State University Tallahassee Florida USA; ^2^ Department of Entomology University of Maryland College Park Maryland USA

**Keywords:** density‐dependent herbivory, density‐dependent survival, distribution of leaf damage, induced resistance, tritrophic interactions

## Abstract

Plant resistance and predators can influence density‐dependent survivorship and growth of herbivores, and their damage to plants. Although the independent effects of plant resistance and predators on herbivores and herbivory are well known, little is known about their interactive and density‐dependent effects on herbivores and the amount and distribution of damage on plants. These relationships are important for understanding how herbivore and plant populations influence each other. We used a laboratory density‐manipulation experiment to determine how plant resistance (three treatments: jasmonate‐insensitive, unmanipulated wild type, and jasmonate‐sprayed wild‐type plants) and predation (two treatments: predator or no predator) affect the survivorship and growth of an herbivore, as well as per capita damage and the distribution of damage on plants. We found evidence that the density dependence of herbivore survivorship was influenced by predators and an interactive effect of plant resistance and predation. Herbivore growth was reduced by higher plant resistance but was not density‐dependent nor affected by predation. Per capita plant damage was reduced by plant resistance, predation, and herbivore density. The within‐plant distribution of damage became more even with increasing herbivore density but was not affected by predation or the independent effect of plant resistance. The distribution of damage was also affected by an interaction between plant resistance and herbivore density; damage became less aggregated with density across all plant resistance treatments, but the decrease was strongest for the jasmonate‐insensitive plants. These results show that predators influence herbivore density dependence, and that plant resistance can affect the impact of predators on herbivores. Though plant resistance, predation, and herbivore density all reduced per capita herbivore damage to plants, only herbivore density and plant resistance affected the distribution of damage. Distributions of herbivory can influence plant success; documenting patterns of herbivory is an under‐appreciated avenue for integrating effects of plant resistance, predators, and herbivore density on plant–herbivore interactions.

## Graphical abstract

Plant resistance and predators can influence survivorship and growth of herbivores, and their damage to plants. We found evidence that the density dependence of herbivore survivorship was influenced by predators and an interactive effect between plant resistance and predation, while herbivore growth was only reduced by plant resistance. Per capita plant damage was reduced by plant resistance, predation, and herbivore density, and plant damage became more even with increasing herbivore density but was not independently influenced by plant resistance or predation.

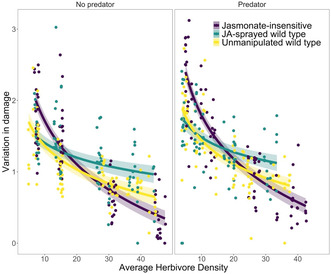

## 1 INTRODUCTION

Many factors are known to influence population sizes of herbivorous insects and the damage that they do to plants. Studies of tritrophic interactions have particularly emphasized that both plants and the predators of herbivores can be influential (Hairston et al., 1960; Price et al., 1980; Stiling, 1988; Vidal & Murphy, 2018), and that plant and predator effects can have statistical interactions (e.g., Kersch‐Becker et al., 2017; Kersch‐Becker & Thaler, 2015; Thaler et al., 2014). Density dependence (the dependence of vital rates, such as survival or growth, on an organism's density) also plays a critical role in insect population dynamics, because the existence and form of density dependence can strongly influence how populations grow and shrink (e.g., Berryman et al., 1987; Turchin, 2003). To understand what controls herbivore populations and their damage, in both natural and managed systems, it is important to determine how all these factors (plants, predators, and herbivore density) interact.

Plant resistance can contribute to density dependence in herbivore populations when resistance is a function of the amount of herbivore damage received by a plant. Plasticity in plant resistance in response to herbivory, or ‘induced resistance,’ is widespread among plants (Karban & Myers, 1989). Many plant traits are associated with induced resistance, including secondary metabolites (Karban & Myers, 1989). The magnitude and/or timing of induced resistance can be dependent on herbivore density and can vary among plant genotypes (Johnson, 2008; Karban & English‐Loeb, 1988; Underwood, 2000, 2010). Previous empirical studies have shown that induced resistance can provide density‐dependent negative feedback to herbivore density (e.g., Karban, 1987; McNutt et al., 2017; Rotem & Agrawal, 2003; Underwood, 2010). However, we know relatively little about how density‐dependent feedback mediated by plant resistance may change when predators are present (but see Kaplan & Thaler, 2010; Kersch‐Becker et al., 2017; Kersch‐Becker & Thaler, 2015).

Predators influence the density dependence of herbivore vital rates through both consumptive (i.e., predation) and non‐consumptive (i.e., perceptions of fear, risk) effects (Kaplan & Thaler, 2010; Preisser et al., 2005). Predators can generate negative density feedbacks in populations of herbivores by foraging in areas of high herbivore density (e.g., Mutz et al., 2020) or through their functional responses (Holling, 1961). Non‐consumptive effects, which occur when the presence of a predator causes an herbivore to change a trait (e.g., behavior, growth) (e.g., Hermann & Thaler, 2014; Schmitz et al., 1997), can also influence herbivore population sizes (Sheriff et al., 2020). Though no study has looked at the consequences of non‐consumptive effects on the density dependence of herbivores, previous studies have shown that both consumptive and non‐consumptive effects of predators can interact with plant resistance, influencing, for example, herbivore vital rates (e.g., growth) and behavior (e.g., feeding; Kaplan et al., 2014; Kaplan & Thaler, 2010).

It seems likely that induced plant resistance and predators should also interact to affect the density dependence of herbivore success (Kersch‐Becker et al., 2017; Kersch‐Becker & Thaler, 2015). Plant resistance can influence the effect of predators on herbivores directly or indirectly (Kaplan & Thaler, 2010). Directly, plant resistance may affect predator efficiency. For example, empirical studies have shown that induced morphological traits (e.g., trichomes, Dalin et al., 2008) can reduce the foraging efficiency of predators (e.g., Riddick & Simmons, 2014). Plant resistance can also increase predator attraction through the release of Herbivore‐Induced Plant Volatiles (HIPVs) (Dicke, 2015; Turlings et al., 1990). Indirectly, plant resistance can influence predators by delaying herbivore development (e.g., Kersch‐Becker et al., 2017; Uesugi, 2015), altering herbivore movement rates (e.g., Agrawal & Karban, 1999; Edwards & Wratten, 1983), or through the sequestration of plant toxins by herbivores to defend themselves from predators (e.g., Camara, 1997). The sequestration of plant toxins by herbivores should decrease the effect of predators on demographic rates. Delays in herbivore development caused by plant resistance should prolong the amount of time that herbivores are vulnerable to predators, thus amplifying the effect of the predators on the demographic rates of herbivores (e.g., Uesugi, 2015). Plant resistance may also increase predation when induced resistance causes herbivores to move away from areas of damage (Agrawal & Karban, 1999; Edwards & Wratten, 1983). However, very few studies have examined density dependence in a tritrophic context (but see Kersch‐Becker et al., 2017; Kersch‐Becker & Thaler, 2015).

The damage that herbivores inflict on plants may be influenced by herbivore density, predation, plant resistance, and their interactive effects. When feeding by herbivores is density‐dependent, the influence of density can be positive (e.g., herbivores facilitate one another's feeding by overcoming leaf toughness or trichomes, overwhelming plant defenses, etc.; Despland, 2019; Ghent, 1960; Inouye & Johnson, 2005), or negative because of intraspecific competition (Karban & Agrawal, 2002). The distribution of damage among the parts of a plant may also be affected by herbivore density (Underwood, 2010), either becoming more even with increasing herbivore density if herbivores avoid each other or previous damage, or less even if herbivores feed together. Plant resistance can influence the distribution of herbivore damage when it causes herbivores to adjust their behavior (Bergelson et al., 1986; Edwards & Wratten, 1983; Rodriguez‐Saona & Thaler, 2005; Underwood et al., 2005). Specifically, theory suggests that induced resistance should cause herbivores to move away from areas of damage or localized resistance and thus cause a more even distribution (Edwards & Wratten, 1983, but see Underwood et al., 2005). Though it is well documented that the effect of predators can also cascade down to influence the mean amount of herbivory (e.g., Schmitz et al., 1997; Thaler & Griffin, 2008), less is known about how predators affect the distribution of damage within host plants. Because predators can alter the behavior of herbivores (e.g., decrease movement or feeding; Bernays, 1997; Thaler et al., 2014), we expect that they might increase aggregation of herbivore damage, concentrating it on certain parts of a plant. To our knowledge, no study has considered the interactive effects of plant resistance and predation on the amount or on the distribution of damage within a plant.

We used a greenhouse experiment with tomato (*Solanum lycopersicum*) and beet armyworm (*Spodoptera exigua*) to examine the interactive effects of plant resistance and predators on the short‐term density dependence of herbivore vital rates (growth and survival), which can influence population dynamics. We also determined how plant resistance, predator presence, and herbivore density might interact to affect plant damage and the distribution of damage within a plant. We used jasmonate‐insensitive plants (JAI‐1 mutant line; non‐plastic, low resistance), unmanipulated wild‐type plants (expected to be plastic, intermediate resistance), and jasmonate elicitor‐sprayed wild‐type plants (hereafter JA‐sprayed wild type; potentially plastic, expected to be highly resistant) crossed with herbivore density treatments and predator treatment (predator and no predator) to ask: (1) Is the density dependence of herbivore survival affected by the independent and/or interactive effects of plant resistance and predation? (2) Is the density dependence of herbivore growth affected by the independent and/or interactive effects of plant resistance and predation? and (3) Are per capita herbivore damage and the distribution of damage within plants affected by the independent effects of and/or interactions among plant defenses, herbivore density, and predators? By manipulating the plasticity of plant defenses, herbivore density, and presence of predators, we contribute to the understanding of how plant resistance and predators jointly influence the population dynamics of herbivores and patterns of herbivory that can influence plant fitness.

## 2 METHODS

We used tomato (*Solanum lycopersicum*), beet armyworm (*Spodoptera exigua*), and spined soldier bugs (*Podisus maculiventris*) as our model tritrophic system. Herbivore damage can induce a variety of changes in tomato foliage, such as increases in the activities of several proteins (e.g., proteinase inhibitors and oxidative enzymes; Stout & Duffey, 1996) and higher trichome densities (Boughton et al., 2005). Induced resistance of tomato has been shown to reduce growth, increase mortality, and deter feeding of herbivores, including *S. exigua* caterpillars (Stout & Duffey, 1996; Thaler et al., 2001). The generalist herbivore, *S. exigua*, is an economically important agricultural pest found globally (Zheng et al., 2011). Typically, *S. exigua* undergoes five instars before pupation. The generalist predator, *P. maculiventris*, feeds on all instars of *S. exigua* (DeClercq & Degheele, 1994).

To manipulate the magnitude and plasticity of plant resistance, we used two lines of tomato plants that differ in their response to jasmonic acid: (1) a wild‐type line known to induce the jasmonate pathway in response to herbivore damage (cv. Castlemart), and (2) a jasmonate‐insensitive line that does not induce in response to damage (cv. JAI‐1; obtained from the J. Thaler lab, Cornell University) that was developed by breeding a JAI‐1 mutation into a Castlemart variety (Li et al., 2001). These two plant lines differ in their production of secondary compounds that are regulated by the jasmonate pathway but otherwise share a common genetic background (Li et al., 2002). By spraying the wild type line with jasmonic acid (JA), we expected to generate higher levels of resistance. In tomato plants, peak chemical resistance occurs 24–48 h after induction and is maintained for weeks (Orians et al., 2000; Stout et al., 1996). We sprayed wild‐type plants with 0.5 mM JA solution 4 days prior to introducing herbivores or predators. Each sprayed plant received 0.47 milligrams of JA; the unmanipulated wild‐type and jasmonate‐insensitive plants were sprayed with the same amount of water to avoid confounding effects of spraying. All tomato plants were germinated in pots containing Fafard® potting soil. Tomato plants were planted in mid‐June 2009 and grown in natural sunlight conditions at the Florida State University greenhouse facility. Seedlings were watered as needed and, once they had two compound leaves, were fertilized every other week.

We maintained a colony of *S. exigua* that was sourced from eggs acquired from Benzon Research Inc. Larvae were reared in a growth chamber (temperature, 28°C; light, 12 L:12D; relative humidity, 75 ± 10%) on *S. exigua* diet (Southland Products Inc). We used adult *P. maculiventris* obtained from a colony that had undergone multiple generations in the lab. All *P. maculiventris* were raised in a growth chamber (temperature, 28°C; light, 12 L:12D; relative humidity, 75 ± 10%) on a diet of fifth‐instar *S. exigua*.

We used a three‐way factorial design to investigate the effects of plant resistance, predator presence, and herbivore density on herbivore survivorship and growth, and plant damage. We combined three plant resistance treatments (JA‐insensitive, unmanipulated wild type, and JA‐sprayed wild type) with two levels of the predator (absent or present), and five levels of initial herbivore density (0, 8, 16, 32, or 48 third‐instar caterpillars) for a total of 30 treatment combinations, each replicated five times. Caterpillars and their damage were measured after 4 days of being exposed to the experimental treatments.

Prior to the start of the experiment, we transplanted tomato plants with three compound leaves into a single Classic 2000 pot (3.84 gallons; Nursery Suppliers, Inc.). Each pot or enclosure contained four plants of the same line. Experimental enclosures (hereafter cages) were created by covering pots with an inverted floral plant sleeve made of breathable material that was secured at the top and sides of a cage. Groups of plants of the wild‐type line were randomly assigned to a resistance treatment (unmanipulated or JA‐sprayed). For logistical reasons, we conducted the experiment in two temporal blocks (block 1: July 17–21, 2009, block 2: July 24–28, 2009). Each temporal block contained at least two replicate cages per treatment combination.

To transition *S. exigua* larvae from a structurally simple environment and artificial diet of a lab colony to a more structurally complex environment and plant diet of the experimental cages, we exposed larvae to tomato leaves for 48 h before the experiment. For the experiment, groups of 8, 16, 32, or 48, third‐instar *S. exigua* were introduced to a cage according to their randomly assigned density treatment. Density treatments were selected based on previous studies that manipulated *S. exigua* density (Smits et al., 1987; Underwood, 2010), and to bracket densities observed in field experiments (Kolodny‐Hirsch et al., 1993). We placed each group of *S. exigua* in an open petri dish that was balanced on the canopy of the tomato plants near the middle of each cage. An hour after *S. exigua* were released into the experimental cages, one adult *P. maculiventris* was added to the predator‐present treatment cages and the empty petri dish was removed. The delay in the addition of *P. maculiventris* was to prevent unnatural predation events because, when disturbed, *S. exigua* will often curl up and remain immobile for a short time.

**FIGURE A1:**
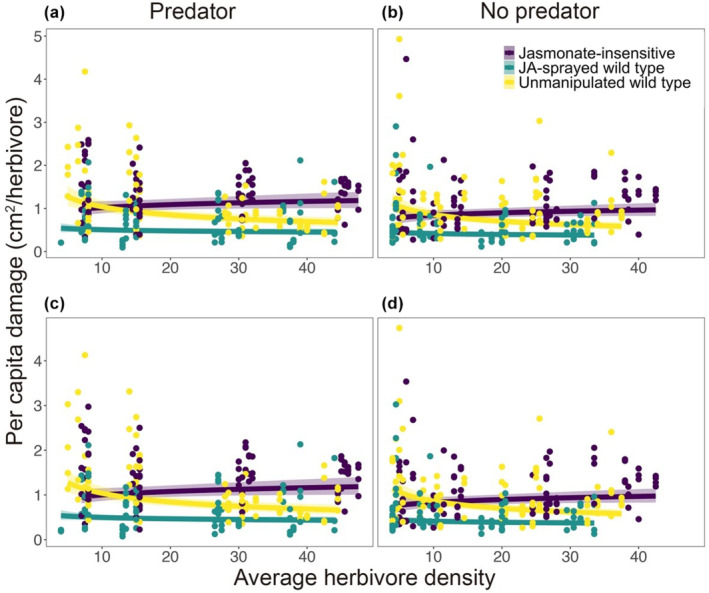
There are no qualitative differences in the effects of herbivore cage‐level density, plant resistance, and predation on per capita damage (cm^2^/herbivore) when leaflets are the same size (a,b) or vary in size (c,d). The two panels on the left (a,c) show the relationship between per capita damage and average herbivore density when a predator was absent, and the two panels on the right (b,d) show the relationship when a predator was present. Plant resistance treatments—JA‐insensitive, unmanipulated wild type, and JA‐sprayed wild type—are indicated by colors: Purple, yellow, and aqua, respectively. The points show the hypothetical per capita damage of herbivores on a single plant with same‐sized leaflets (a,b) or different‐sized, randomly assigned leaflets (c,d). Though we used linear mixed models with cage as a random effect, the lines and shaded regions correspond to the predictions and 95% CI of linear models with the same structure but without the random effect, to facilitate plotting.

**FIGURE A2:**
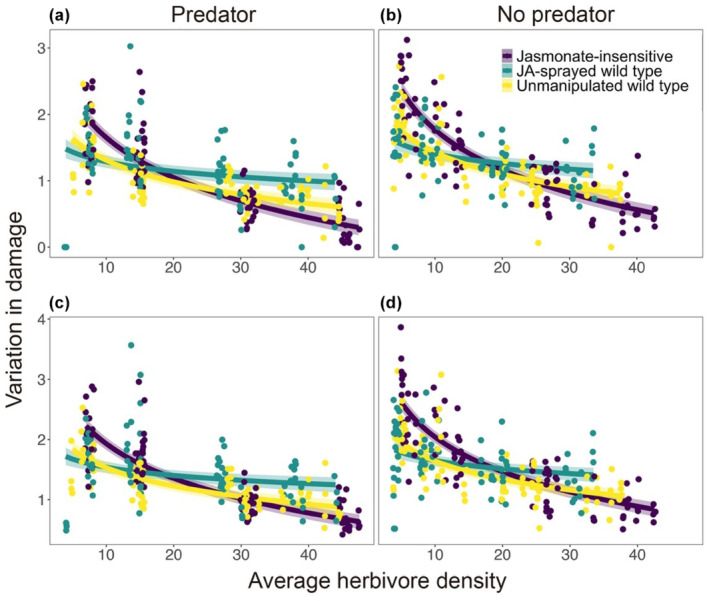
There are no qualitative differences in the effects of herbivore cage‐level density, plant resistance, and predation on variation in damage when leaflets are the same size (a,b) or different sizes (c,d). The two panels on the left (a,c) show the relationship between variation in damage and average herbivore density when a predator was absent, and the two panels on the right (b,d) show the relationship when a predator was present. Colors correspond to plant resistance treatments: Purple represents JA‐insensitive, yellow shows unmanipulated wild type, and aqua shows JA‐sprayed wild type. The points show the simulated variation in damage of herbivores on a single plant with same‐sized leaflets (a,b) or different‐sized, randomly assigned leaflets (c,d). Though we used linear mixed models with cage as a random effect, the lines and shaded regions correspond to the predictions and 95% CI of linear models with the same structure but without the random effect, to facilitate plotting.

To determine the effect of plant resistance and predator treatment on *S. exigua* performance, we counted the number of larvae alive following 4 days in the experiment cages and scored the developmental stage of each survivor to determine survivor growth rate. Third‐, fourth‐, and fifth‐instar caterpillars were scored 3, 4, and 5, respectively, while pupae received a score of 6. We were able to determine the number of caterpillars eaten by the predator, *P. maculiventris*, by counting the empty exoskeletons of caterpillar prey. All tomato plants and cage surfaces were inspected thoroughly, and the top layer of soil in the pots was sieved to locate surviving *S. exigua*. All caterpillars were accounted for. To characterize plant damage, we visually inspected all leaflets in the enclosures and assigned every leaflet to one of five damage categories based on the percentage of the leaflet that was damaged: 0% – 2%, 3% – 25%, 26% – 50%, 51% – 75%, and 76% – 99%. We recognize that this method can underestimate (on larger leaflets) or overestimate (on smaller leaflets) relative leaflet damage if leaves vary in size. To ensure that size variation among leaflets did not influence our results, we performed a simulation where we estimated absolute damage using randomly assigned leaflet areas (see Appendix A for detailed methods). Results from the simulation show that percent damage does not influence our conclusions (Figures A1 and A2 in Appendix A). We used the midpoints of damage categories (1%, 14%, 38%, 63%, or 88%) to calculate mean leaflet damage for each plant, which served as an estimate of total damage at the plant level. We subsequently divided the total damage estimate for each plant by the mean of the initial and final herbivore density (i.e., average density) in its enclosure, to calculate a measure of damage per herbivore for use in statistical analyses. We used per capita damage instead of total plant damage to capture differences in the feeding rates of individuals in response to plant resistance, predator treatment, and herbivore density. We measured the distribution of damage as the coefficient of variation (CV) of leaflet damage.

### 2.1 Protease inhibitor assay

To determine how the plant resistance treatments affected one potential mechanism of plant resistance (unmeasured mechanisms could include trichomes, leaf toughness, and other aspects of leaf chemical content and nutritional quality), we measured the activity of trypsin protease inhibitor (PI) (see Appendix B for detailed methods). Briefly, we measured trypsin PI activity in undamaged leaflets from each plant by clipping a leaflet at the start and end of the experiment. The clipping of a leaflet at the start of the experiment is unlikely to have induced resistance against the herbivores; previous studies have shown that plants respond differently to mechanical damage, such as clipping, compared to herbivore damage (e.g., Agrawal & Sherriffs, 2001; Baldwin, 1990). Due to logistical constraints, assays were only performed for cages without predators.

### 2.2 Statistical analysis

All analyses were done using R 4.2.2 (R core team, 2022). To evaluate how plant resistance and the presence of a predator influenced the density dependence of survival and growth of herbivores, we used linear models (Gaussian distribution) with each vital rate as a response variable. We estimated survival as the proportion of surviving *S. exigua* within each cage (i.e., survivorship). Herbivore growth was estimated as the mean instar score of surviving *S. exigua* within each cage. We excluded cages from the herbivore growth model that had no surviving *S. exigua*. We started with full models for each vital rate that included average herbivore density through the experiment, plant resistance (JA‐insensitive, unmanipulated wild type, or JA‐sprayed wild type), predator treatment (present or absent), and temporal block as predictor variables. We used average herbivore density (mean of initial and final density, a continuous variable) rather than just initial density (a categorical variable) to account for changes in density within cages throughout the course of the experiment. We used a backward step‐wise approach to compare all possible combinations of additive and interactive effects (two‐way and three‐way interactions) among herbivore density, plant resistance, and predator treatment. To compare fits among models, we used Akaike's Information Criterion (AIC; ‘AICcmodavg’ package) and retained the model with greatest support. We then used a type II ANOVA for hypothesis testing (‘car’ package); we acknowledge that this workflow does not fully control for type II error rates but believe it aids in identifying potentially important biological process. We checked that our residuals met model assumptions of normality, homoscedasticity, and independence. Average density was log‐transformed to linearize the relationship between it and the response variables. All results are presented for back‐transformed variables. Two cages (cage 1 treatment: JA‐sprayed wild type, initial density of 48 herbivores, and predator present; cage 2 treatment: JA‐sprayed wild type, initial density of eight herbivores, and no predator) were removed from all statistical analyses due to human error; no herbivores were added to the first cage, and data were missing for the second cage. There were seven cages where no herbivores survived, and these were removed from the model with herbivore growth as a response variable (all seven cages had predators; three cages were JA‐sprayed wild type with initial density of eight herbivores, two cages were JA‐sprayed wild type with initial density of 16 herbivores, and two cages had unmanipulated wild‐type plants with eight and 16 herbivores at the start of the experiment).

**FIGURE C1:**
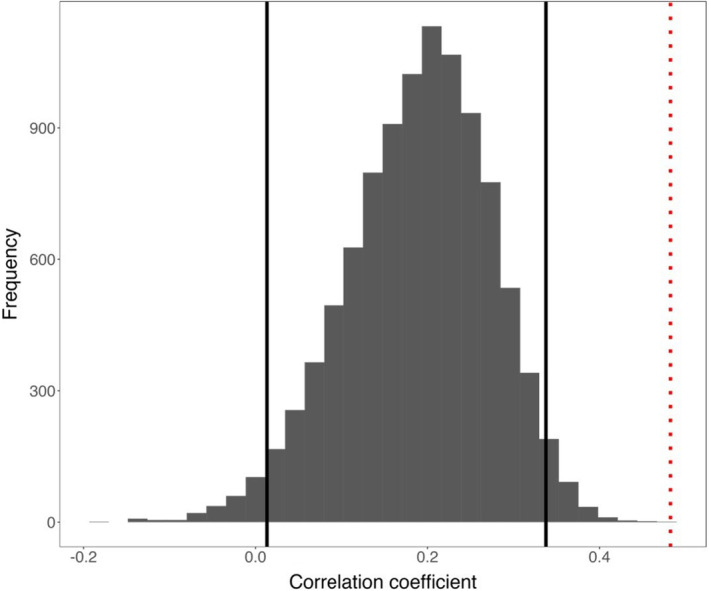
The observed correlation between average herbivore density and per capita damage is in the upper 5th percentile of the distribution of correlation coefficients. We checked for spurious correlations between per capita damage (i.e., the quotient of mean damage per plant and average herbivore density) and average herbivore density by creating lognormal distributions for each cage using the mean and standard deviation of per capita damage and the mean and standard deviation of mean damage (i.e., mean amount of plant damage not divided by average herbivore density). We calculated a correlation coefficient across all cages by calculating the correlation between a randomly drawn per capita damage and a randomly drawn transformed mean damage (i.e., randomly drawn mean damage divided by average herbivore density). We performed 10,000 iterations for a total of 10,000 correlation coefficients. The solid black lines indicate the lower and upper 5th percentiles, which are the most negative and positive correlations, respectively, that can arise by chance when there is no correlation between the two variables. The dashed red line represents the observed correlation between average herbivore density and per capita damage.

To determine the effects of plant resistance, average herbivore density, and predator treatment on both mean amount of damage per herbivore and the distribution of damage, we used linear mixed effects models (‘lme4’ package; Gaussian distribution). Though there are several levels of organization (e.g., leaf, plant, and cage) at which we could analyze per capita damage and distribution of damage, we conducted these analyses at the scale of the plant (i.e., mean amount of damage and distribution of damage across leaflets within a plant). We determined the distribution of damage using the coefficient of variation (CV) in damage among leaflets of a plant. We assigned log average density, plant resistance, predator treatment, and block as fixed effects. Spurious correlations can occur in a model when a variable is regressed onto itself, as in the case of our linear mixed effects model where average herbivore density is both a predictor and part of the response variable (i.e., amount of damage divided by average herbivore density). To check for a spurious correlation in our model, we used the method described by Morris et al. (2006). The observed correlation is above the *p*‐value threshold indicating that our results are not driven by an interdependence between average herbivore density and per capita damage (Appendix C, Figure C1). To account for repeated measures within cages, the unique ID of the plant's cage was included as a random effect. To meet model assumptions, we applied a one‐third power transformation to mean damage. To account for more precise measures of the CV among plants with more leaflets, we included the square root of the number of leaflets as a weighting term in the model. We used a backward step‐wise approach to test combinations of additive and interactive effects among plant resistance, average herbivore density, and predation treatments. We compared the fits of models using Akaike's Information Criterion (AIC; ‘AICcmodavg’ package) and then a type II ANOVA for hypothesis testing (‘car’ package).

To characterize one potential mechanism of resistance and change in resistance in response to damage in our three plant resistance treatments, we used mixed effects models with mean PI values (mean of starting and ending PI values) and ending PI values (PIs after damage) as response variables. Both models included plant resistance treatment, average herbivore density (mean of the beginning and ending herbivore densities), and block as predictors. We also included a two‐way interaction between plant resistance and average herbivore density. We included cage ID as a random effect, as we had measurements from multiple plants within each cage. We again compared the fits of the full models (i.e., with the two‐way interaction) to reduced models (i.e., without the two‐way interaction) using Akaike's Information Criterion (AIC; ‘AICcmodavg’ package) and then a type II ANOVA for hypothesis testing (‘car’ package).

## 3 RESULTS

### 3.1 Questions 1 and 2: the independent and interactive effects of plant resistance and predators on the density dependence of herbivore survivorship and growth

**FIGURE 1:**
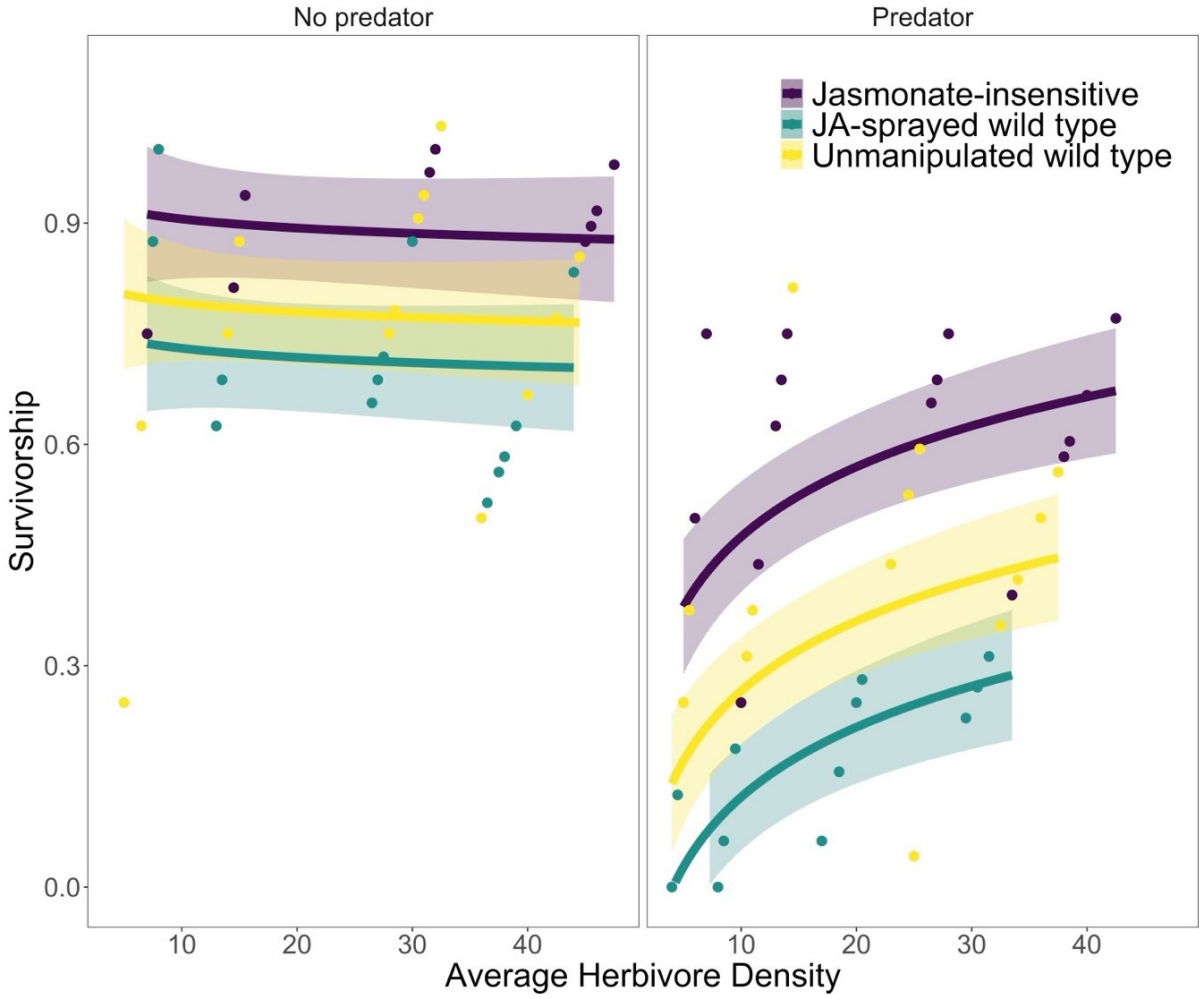
The proportion of surviving *S. exigua* varied with cage‐level average herbivore density (mean of initial and final herbivore density), the presence of a predator, and plant resistance treatment. The effect of plant resistance on survivorship was tested using three resistance levels: JA‐insensitive (low resistance; purple), unmanipulated wild type (with induced resistance; yellow), and JA‐sprayed wild type (consistently resistant; aqua). Points show the proportion of surviving herbivores in each cage. Lines and shaded regions correspond to predicted survivorship and 95% confidence intervals of the best‐supported linear model. The two panels show survivorship without (right panel) and with (right panel) one adult *P. maculiventris*.

The best‐fit model for herbivore survivorship retained additive terms for all three focal predictors (plant resistance, predators, and average herbivore density) along with two interaction terms involving: (1) plant resistance and predators, and (2) average herbivore density and predators. Survival of *S. exigua* was negatively density‐dependent (*F*_1,109_ = 13.8, *p* = .0003, Figure 1; Table 1). Plant resistance had a significant effect on survivorship (*F*_2,109_ = 33.2, *p* < .0001). On average, a higher proportion of herbivores survived in the JA‐insensitive treatment compared to the unmanipulated wild type or JA‐sprayed wild type (mean ± SE; JA‐insensitive, 0.74 ± 0.04; unmanipulated wild type, 0.56 ± 0.05; JA‐sprayed wild type, 0.45 ± 0.05). On average, survivorship significantly decreased in treatments with predators (*F*_1,109_ = 264.9, *p* < .0001; mean ± SE; predator present, 0.35 ± 0.03; predator absent, 0.82 ± 0.02). There was a significant interaction between predation and average herbivore density on survivorship *(F*_1,109_ = 17.2, *p* < .0001); survivorship increased with average herbivore density in treatments with predators but was nearly density‐independent when predators were absent (Figure 1). There was also a significant interaction between plant resistance and predator treatments (*F*_*2*,109_ = 3.72, *p* = .027). The difference in survivorship between predation and no predation treatments was greater in the unmanipulated wild type (predator present, 0.33 ± 0.05; predator absent, 0.80 ± 0.04) and JA‐sprayed wild type (predator present, 0.16 ± 0.03; predator absent, 0.74 ± 0.03) compared to the JA‐insensitive treatment (predator present, 0.56 ± 0.04; predator absent, 0.91 ± 0.01). There was no difference in the proportion of surviving *S. exigua* between the two temporal blocks (*F*_*1*,109_ = 1.14, *p* = .288).

**TABLE 1:** Summary of results for all four response variables. Statistical tests are provided only for terms retained after step‐wise AIC model selection.

Predictor	Survivorship		Growth		Per capita damage		Variation in damage	
	F‐value_df_	*p*‐value	F‐value_df_	*p*‐value	*χ* ^2^‐value_df_	*p*‐value	*χ* ^2^‐value_df_	*p*‐value
Average density	**13.8**_**1,109**_	**.0003**	1.55_1,106_	.22	**7.3**_**1**_	**.007**	**289.3**_**1**_	**<.0001**
Plant resistance	**33.2**_**2,109**_	**<.0001**	**50.1**_**2,106**_	**<.0001**	**109.2**_**2**_	**<.0001**	5.74_2_	.057
Predator	**264.9**_**1,109**_	**<.0001**	NA	NA	**11.9**_**1**_	**.0006**	2.5_1_	.114
Plant resistance*Predator	**3.72**_**2,109**_	**.027**	NA	NA	NA	NA	NA	NA
Plant resistance*Density	NA	NA	NA	NA	**14.7**_**2**_	**.0006**	**55.2**_**2**_	**<.0001**
Predator*Density	**17.2**_**1,109**_	**<.0001**	NA	NA	NA	NA	NA	NA
Block	1.14_1,109_	.288	**21.8**_**1,106**_	**<.0001**	**13.9**_**1**_	**.0002**	0.087_1_	.77

*Note*: F‐values and degrees of freedom are shown for survivorship and growth. For mean damage and variation in damage, *X* ^*2*^ and degrees of freedom are shown. *p*‐Values are shown for all terms under the F‐ or *X* ^*2*^‐values; terms with *p*‐values <.05 are in bold.

**FIGURE 2:**
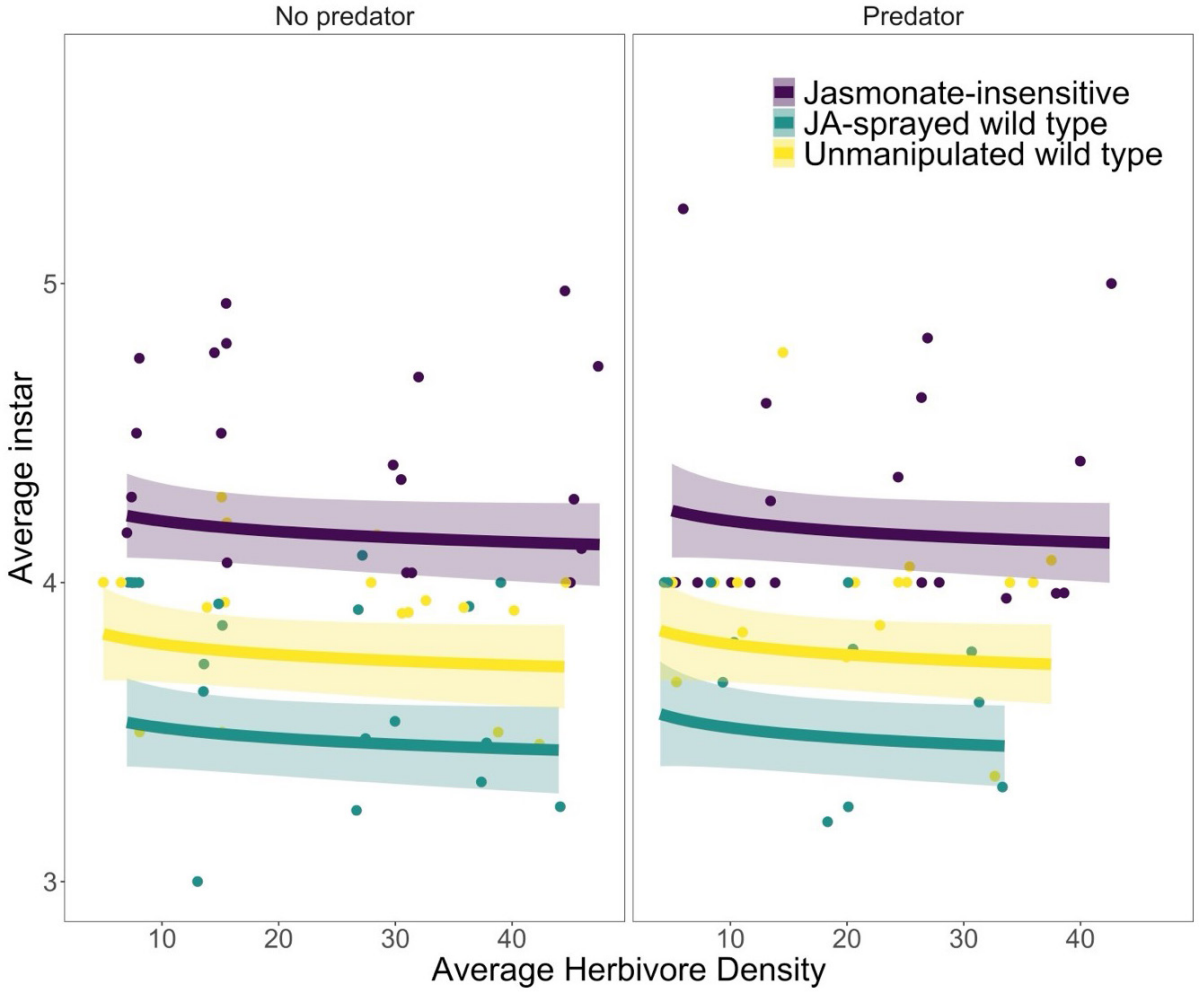
The average instar of surviving *S. exigua* in each cage varied with plant resistance treatment across cage‐level average herbivore density (mean of initial and final herbivore density). The absence (left panel) or presence (right panel) of a predator had no effect on average instar. Colors correspond to plant resistance treatments: Purple represents JA‐insensitive, yellow shows unmanipulated wild type, and aqua shows JA‐sprayed wild type. Each point shows the average instar of a cage. The lines and shaded regions show predictions and 95% confidence intervals from the linear model.

For herbivore growth, the best‐fit model only retained two predictors: plant resistance and average herbivore density. The average instar of surviving *S. exigua* was not significantly affected by conspecific density (*F*_1,106_ = 1.55, *p* = .22; Figure 2; Table 1) but was significantly affected by plant resistance (*F*_2,106_ = 50.1, *p* < .0001). On average, *S. exigua* reached a higher instar in the JA‐insensitive (mean ± SE; 4.3 ± 0.06 instar) treatment compared to the unmanipulated wild type (3.9 ± 0.04 instar) and JA‐sprayed wild type (3.7 ± 0.06 instar) treatments. Temporal block was retained in the best model, and larvae grew more, on average, in the second block (*F*_1,106_ = 21.8, *p* < .0001).

### 3.2 Question 3: are mean herbivore damage and the dispersion of damage within plants affected by the interactions among plant resistance, herbivore density, and predators?

**FIGURE 3:**
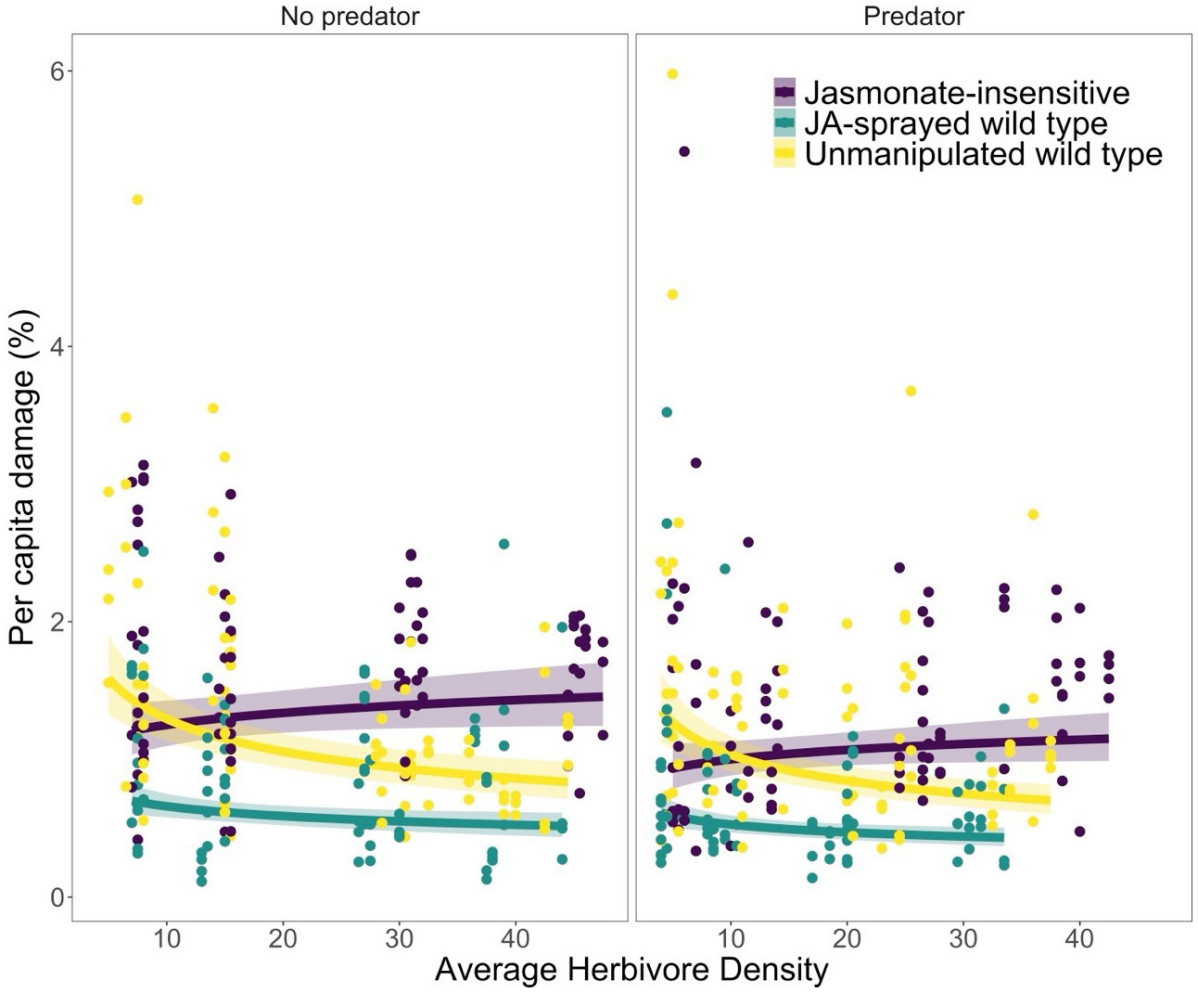
Per capita damage (mean leaflet percent damage per herbivore) was influenced by herbivore cage‐level density (mean of initial and final herbivore density), plant resistance, predator presence, and an interaction between plant resistance and herbivore density. The two panels show the relationship between mean damage and average herbivore density when a predator was absent (left panel) or present (right panel). Colors—purple, yellow, and aqua—indicate plant resistance treatments: JA‐insensitive, unmanipulated wild type, and JA‐sprayed wild type, respectively. The points show per capita damage of herbivores on a single plant. Though we used linear mixed models with cage as a random effect, the lines and shaded regions correspond to the predictions and 95% CI of linear models with the same structure but without the random effect, to facilitate plotting.

The best‐fit model for per capita herbivore damage included additive effects of average herbivore density, plant resistance, predation, and temporal block, and retained an interaction term between average herbivore density and plant resistance. There was a significant effect of *S. exigua* density on per capita damage (Density: *χ* ^2^_*1*_ = 7.3, *p* = .007; Figure 3; Table 1) but the direction of the effect varied with plant resistance treatment (i.e., interaction between average density and plant resistance; *χ* ^2^_*2*_ = 14.7, *p* = .0006). Per capita damage decreased with average herbivore density in the JA‐sprayed wild type and unmanipulated wild type relative to the JA‐insensitive treatment (Figure 3). Plant resistance significantly affected the average amount of per capita damage (*χ* ^2^_*2*_ = 109.2, *p* < .0001). On average, herbivores inflicted more damage on plants in the JA‐insensitive treatment (mean ± SE; 1.5 ± 0.06% leaflet removed per herbivore) than plants in the unmanipulated wild type (1.4 ± 0.07% leaflet removed per herbivore) or JA‐sprayed wild type (0.8 ± 0.05% leaflet removed per herbivore) treatments. Predators decreased the average amount of damage per herbivore (*χ* ^2^_*1*_ = 11.9, *p* = .0006; mean ± SE; predator present, 1.2 ± 0.05% leaflet removed per herbivore; predator absent, 1.3 ± 0.05% leaflet removed per herbivore). There was a significant effect of temporal block on per capita damage (*χ* ^2^_*1*_ = 13.9, *p* = .0002).

**FIGURE 4:**
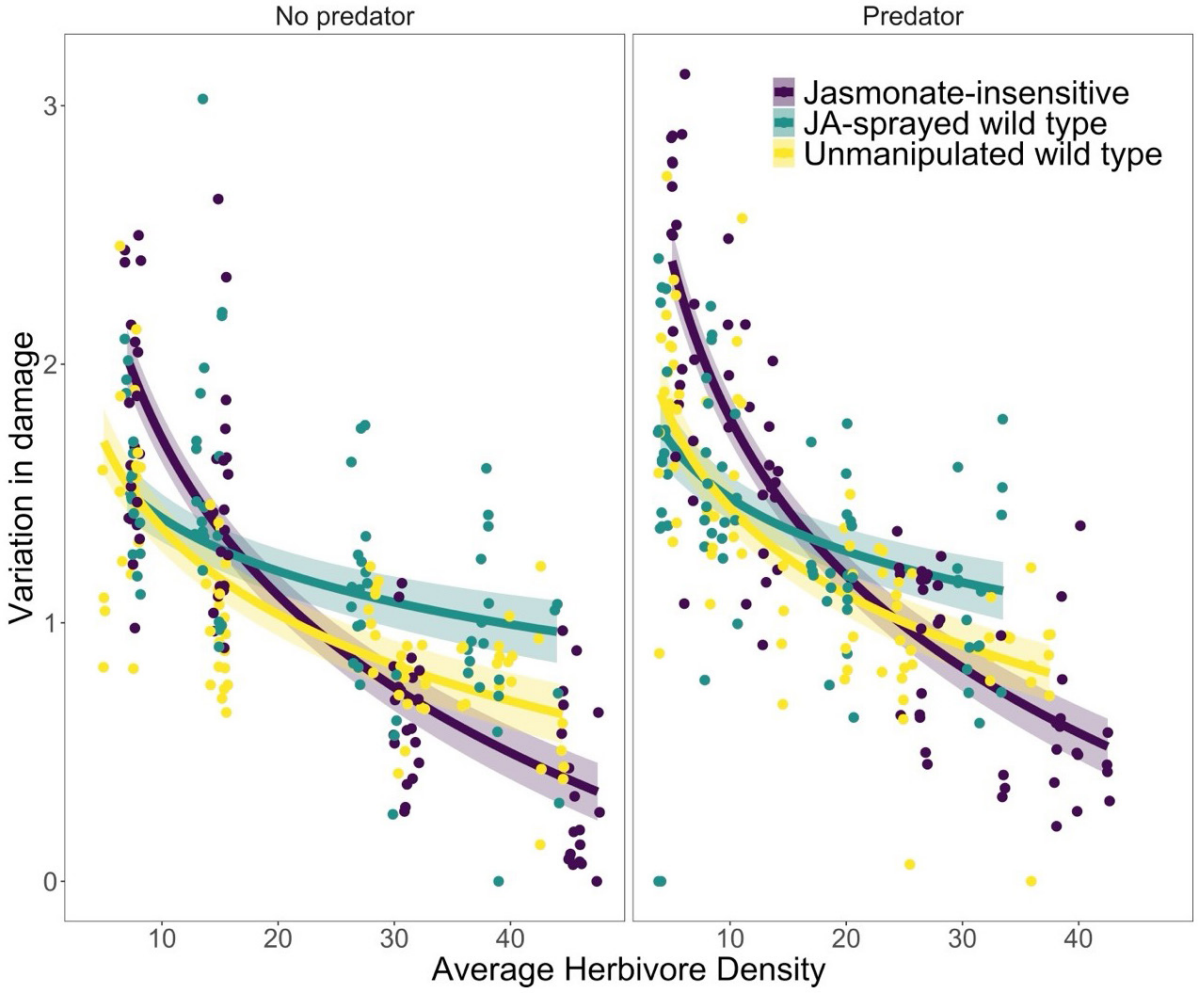
Variation in the distribution of damage within a plant was affected by herbivore cage‐level average density (mean of initial and final herbivore density) and an interaction between plant resistance and predation. The two panels show results when a predator was not present (left panel) or present (right panel). Colors—purple, yellow, and aqua—indicate plant resistance treatments: JA‐insensitive, unmanipulated wild type, and JA‐sprayed wild type, respectively. Each point represents the coefficient of variation (CV) in damage among leaflets within a single plant. Here, we show the results of a linear model with the same structure (except for the random effect of cage) as our mixed effect model. The lines and shaded regions show predicted variation in damage and 95% CI.

The best‐supported model for herbivore damage dispersion retained plant resistance, predator treatment, herbivore density, temporal block, and an interactive effect of plant resistance and herbivore density. The CV of damage significantly decreased with increasing density of *S. exigua* (*χ* ^2^_*1*_ = 289.3, *p* < .0001; Figure 4; Table 1), indicating a more even distribution of damage among leaflets at higher densities. Plant resistance had a marginally significant effect on CV of damage (*χ* ^2^_*2*_ = 5.74, *p* = .057). There was more variation in leaflet damage in the JA‐sprayed (mean ± SE; 1.3 ± 0.04) than the JA‐insensitive (1.2 ± 0.06) and unmanipulated wild‐type treatments (1.2 ± 0.04). The effect of plant resistance interacted with herbivore density (*χ* ^2^_*2*_ = 55.2, *p* < .0001), with CV increasing more with density in the unmanipulated wild‐type and JA‐sprayed treatments relative to the JA‐insensitive treatment (Figure 4). The presence of a predator did not influence CV of damage (*χ* ^2^_*1*_ = 2.5, *p* = .114; mean ± SE; predator present, 1.3 ± 0.04; predator absent, 1.1 ± 0.04). Damage dispersion did not differ between temporal blocks (*χ* ^2^_*2*_ = 0.087, *p* = .77).

### 3.3 Trypsin PI assays

**FIGURE B1:**
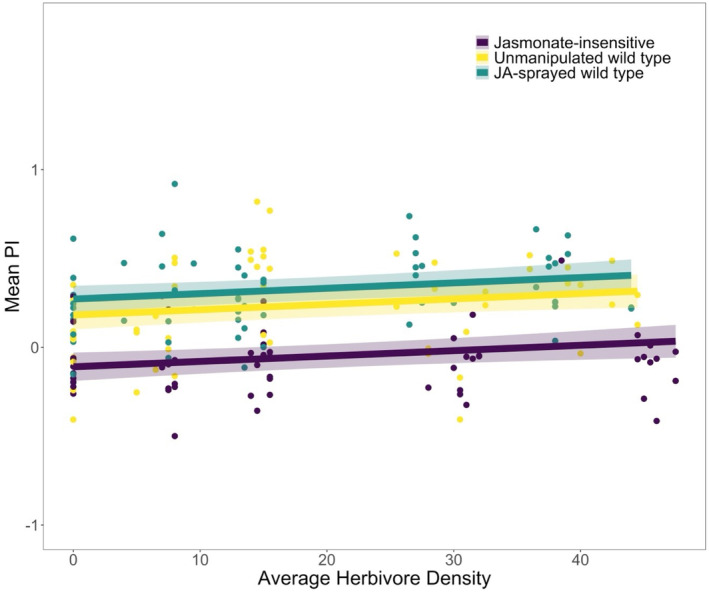
The average PI level of a plant during the experiment differed was influenced by plant resistance and average herbivore density. Three resistance treatments were used: Jasmonate‐insensitive (i.e., low resistance, no induction; purple), unmanipulated wild type (i.e., induced resistance; yellow), and JA‐sprayed wild type (i.e., high resistance with some induction; aqua). We measured the PI levels of two plants in each predator‐free cage at the start and end of the experiment. The two measurements were used to calculate the average PI. The points represent the mean PI levels of plants throughout the experiment. Though a mixed effects model that included a random effect of cage was used for analysis, we show here a linear model with the same structure sans the random effect. The lines and shaded regions show predictions from a linear model that included plant resistance, average herbivore density, and block as additive predictors.

**FIGURE B2:**
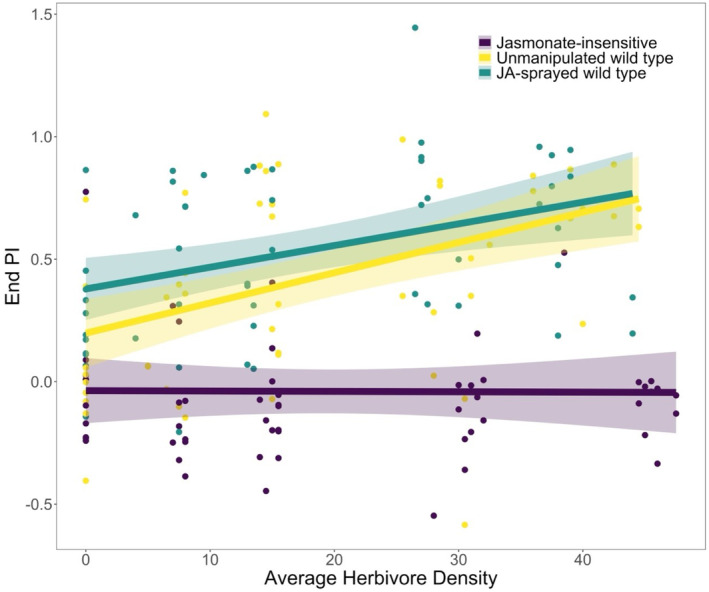
PI levels at the end of the experiment were influenced by plant resistance treatment and average herbivore density. Three resistance treatments were used: Jasmonate‐insensitive (i.e., low resistance, no induction; purple), unmanipulated wild type (i.e., induced resistance; yellow), and JA‐sprayed wild type (i.e., high resistance with some induction; aqua). The points represent the PI level of a plant at the end of the experiment. Though we used a mixed effects model that included random effect of cage for analysis, we show here a linear model with the same structure but without the random effect to facilitate plotting. The lines and shaded regions represent predictions from a linear model that included plant resistance treatment, average herbivore density, an interaction between plant resistance and average herbivore density, and block as predictors.

Mean PI levels differed among plant resistance treatments (*χ* ^2^_*2*_ = 77.9, *p* < .0001; Appendix B, Figure B1) and with average herbivore density (*χ* ^2^_*1*_ = 6.21, *p* = .013) according to the best‐supported model. There was no difference in PI levels between the two temporal blocks (*χ* ^2^_*1*_ = 2.2, *p* = .134). Overall, mean PI levels were lower in the JA‐insensitive treatment (mean ± SE; −0.08 ± 0.03) than the two wild‐type treatments (mean ± SE; unmanipulated wild type, 0.21 ± 0.04; JA‐sprayed wild type, 0.30 ± 0.03; Appendix B, Figure B1), which did not differ from each other (CIs overlap). The PI levels at the end of the experiment were influenced by average herbivore density (*χ* ^2^_*1*_ = 14.7, *p* = .0001; Appendix B, Figure B2), plant resistance treatment (*χ* ^2^_*2*_ = 99.6, *p* < .001), and an interaction between these two predictors (*χ* ^2^_*2*_ = 8.96, *p* = .01). There was no effect of temporal block on end PI levels density (*χ* ^2^_*1*_ = 2.44, *p* = .118). On average, PI levels were lowest at the end of the experiment in the JA‐insensitive treatment (mean ± SE; −0.08 ± 0.03), followed by the unmanipulated wild‐type (0.38 ± 0.05) and JA‐sprayed wild‐type (0.5 ± 0.05) treatments.

## 4 DISCUSSION

We used a greenhouse experiment to investigate the interactive effects of plant resistance and predators on the density dependence of an herbivore's vital rates and patterns of herbivory. We found that survivorship of herbivores was influenced by two interactions. First, the effect of predators on herbivore survivorship depended on herbivore density; the density‐dependent effect of predators was greater (i.e., lower survivorship) at low herbivore densities (Figure 1). Second, plant resistance modified the interaction between herbivore survivorship and predators, or vice versa. We also found evidence of an interaction between plant resistance type and herbivore density on the distribution of damage, indicating that the effect of plant resistance on the distribution of damage is dependent on herbivore density. Overall, our results suggest that it is important to consider the effects of both plant resistance and predators when evaluating density‐dependent vital rates of herbivore populations and their damage to plants.

The effects of plant resistance type on *S. exigua* growth and survival were a mix of expected and unexpected results. Previous studies have shown that tomato resistance can reduce herbivore performance (e.g., Stout & Duffey, 1996; Thaler et al., 2001; Underwood, 2010) and increase rates of cannibalism (Orrock et al., 2017). As expected, we found that *S. exigua* survival and growth (average instar of survivors) were higher on jasmonate‐insensitive mutants, which had both lower mean PI and end PI. We expected sprayed wild‐type plants to have higher resistance than unmanipulated wild types. Instead, we observed no significant difference in mean PI or herbivore performance (see Figures 1 and 2) between the unmanipulated and JA‐sprayed wild types (i.e., confidence intervals overlapped), although sprayed plants tended to have higher PIs than unsprayed wild types (see Appendix B, Figures B1 and B2). Although the two wild‐type treatments behaved similarly according to the PI assays (Appendix B, Figures B1 and B2), we analyzed them as separate treatments because trypsin PIs are only one facet of a tomato plant's JA‐induced resistance (Thaler et al., 1996). Plant resistance type affected survivorship and growth, but the effect of plant resistance was the same across all herbivore densities; in the absence of predators *S. exigua* survival and growth were not herbivore density‐dependent. This was not expected, based on previous work that has shown inducible resistance in tomato variety Castlemart (e.g., Kersch‐Becker et al., 2017; Underwood, 2010), and our finding that trypsin PI levels after damage (i.e., end PI levels; Appendix B, Figure B2) did increase with herbivore density for unsprayed and sprayed wild‐type plants but not for the jasmonate‐insensitive mutants. Studies conducted over longer time periods relative to the herbivore's life span have found significant herbivore density‐dependent effects of induced plant resistance (e.g., Kersch‐Becker & Thaler, 2019), suggesting that the lack of density‐dependent effect in our experiment may be in part due to the short time span of our experiment (4 days).

Predators reduced herbivore survivorship in this study, and the effect of predators on herbivore survivorship was herbivore density‐dependent. Though predators can have non‐consumptive effects, such as reducing the feeding activity of herbivores, the lack of difference in the average instar of herbivores in treatments with and without predators suggests that changes in feeding behavior with size were insufficient to affect herbivore growth or survival. Alternatively, reduced feeding on plants in treatments with predators may have been offset by cannibalism (Orrock et al., 2017). An increase in the occurrence of cannibalism aligns with the observed effects of predation: lower survivorship (Figure 1), lack of effect on growth (Figure 2), and reduced mean damage levels (Figure 3). We observed that survival decreased slightly with increasing herbivore density in predator‐free treatments, likely because of intraspecific competition. In contrast, in the presence of a predator, survival increased with increasing herbivore density. Survivorship may have increased with density because of predator satiation (e.g., predators reached a limit on handling time or attack rate). The results of our experiment are consistent with other lab‐based empirical studies showing increased survival rates with increasing prey density when predators are present (Aqueel & Leather, 2012; Kersch‐Becker et al., 2017).

Though on average plant resistance did not influence density‐dependent survivorship of *S. exigua*, we found evidence of an interaction between plant resistance and predation, suggesting that plant resistance may influence density‐dependent survivorship by altering the impact of predators. We propose that plant resistance modified the impact of predators, and not vice versa, because of the various mechanisms through which plant resistance has been shown to affect predator–herbivore interactions, such as reducing the performance of predators, influencing prey quality (including prey size), and reducing herbivore‐induced plant volatiles (HIPVs) (Kaplan & Thaler, 2010; Kersch‐Becker et al., 2017). Though our experiment did not test explicitly for a mechanism, our results are most consistent with plant resistance reducing prey quality. Specifically, plant resistance may have influenced the interaction between *S. exigua* and *P. maculiventris* by reducing the size of herbivores (using average instar as a proxy; Figure 2) through delaying development. Predators might thus have compensated through increased predation rates, leading to lower herbivore survivorship (Figure 1). The other proposed mechanisms are less consistent with our results; a reduction in predator performance, HIPVs, or herbivore toxin sequestration should all correspond to higher survivorship (or lower predation rates) in treatments with plant resistance. Our results are consistent with the only other study that has assessed the interactive effects of plant resistance and predation on an herbivore population's density‐dependent vital rates (population growth). Kersch‐Becker et al. (2017) showed that plant resistance can indirectly alter the density‐dependent population growth of an insect herbivore by increasing the abundance and diversity of predators in the field, and by altering prey quality in the lab when predators are given no choice in prey (as in our experiment).

Per capita leaflet damage was affected by herbivore density, plant resistance, and the presence of a predator. Our best‐supported model indicated a marginally significant nonlinear relationship between density and per capita damage. Specifically, the model suggested that per capita damage decreased initially with increasing herbivore density, which is consistent with a negative feedback between induced resistance and plant damage. At higher densities, however, per capita damage seems to level off or increase slightly with increasing herbivore density. A possible mechanism is that increased density may lead more quickly to the damage threshold required to induce resistance and/or maximum resistance (i.e., damage above this threshold does not elicit more induced resistance; Underwood, 2010), and herbivores compensate for poor plant quality by eating more. Overall, per capita damage decreased with herbivore density, suggesting intraspecific competition and/or a negative feedback between plant resistance and herbivore density. There was a significant effect of plant resistance on per capita damage, which is consistent with other many empirical studies where plant resistance has been shown to reduce overall herbivory (e.g., Edwards & Wratten, 1983). As expected, we also observed reduced damage when a predator was present.

The within‐plant CV of leaf damage was influenced by herbivore density. As expected, and consistent with other studies (e.g., Underwood, 2010), increasing herbivore density reduced variation in damage (i.e., damage was more evenly distributed throughout plants at higher herbivore densities). Though previous studies have argued that plant resistance may decrease variation in damage because it causes foraging herbivores to move around more (e.g., Rodriguez‐Saona & Thaler, 2005), our results suggest that the effect of plant resistance on the distribution of damage depends on herbivore density. At low densities, sprayed and wild‐type plants had less variation in damage (i.e., a more even distribution of damage) relative to JA‐insensitive treatments (Figure 4), which is consistent with the idea that induced resistance causes herbivores to move more within a plant (Edwards & Wratten, 1983). At higher densities, however, the sprayed and wild‐type treatments have higher CV values than the JA‐insensitive treatment (Figure 4). Though additional experiments are needed to identify a mechanism, we suggest that the threshold of damage required to induce resistance may have been reached more rapidly at higher densities. Theory suggests that herbivores can become aggregated, increasing variation in plant damage, when there is a threshold of damage required to induce resistance (Anderson et al., 2015). While our results suggest that within‐plant CV of leaflet damage is not affected by predation, it is possible that the threat of predation was not enough to induce behavioral changes (e.g., reduced feeding or movement) among the herbivores.

## 5 CONCLUSIONS

Our study shows that plant resistance and predation may interact to influence the density‐dependent survival of herbivores and their growth rates. Though we found no evidence of an interaction between plant resistance and herbivore density for herbivore survival, the other interactions we observed (plant resistance and predation, predation and herbivore density) suggest that plants can affect density‐dependent survival of herbivores by altering predator–herbivore interactions, as shown in Kersch‐Becker et al. (2017). Additional experiments are needed to test mechanisms, but the reduced survivorship of herbivores when predators are present suggests that induced plant resistance may alter the quality of herbivores, forcing predators to compensate by consuming more prey.

Importantly, our results suggest that studies of plant–insect interactions should consider the density dependence of the distribution of damage in addition to the mean. Consistent with the one other study that has addressed their interactions, we found that herbivore density, plant resistance, and predator presence all influenced the mean per capita damage inflicted by herbivores, which may affect the strength of the negative feedback between plant resistance and herbivore density. We also showed, for the first time, that within‐plant variation in leaf damage is affected by herbivore density, as well as an interaction between plant resistance and herbivore density. Because the within‐plant dispersion of damage can influence plant fitness (Marquis, 1992; Mauricio et al., 1993), our results reveal multiple reasons why it is important to consider herbivore density, plant resistance, higher trophic levels, and interactions among these factors and others when investigating patterns of plant damage.

Our three‐way factorial experiment allowed us to examine many possible interactions in this tritrophic system. Looking at each factor alone would miss potentially important drivers of plant–insect interactions, assuming that the short‐term rates measured in this study scale up to influence longer‐term behavior or population dynamics. Consistent with previous calls for more consideration of variance in ecological systems, rather than focusing solely on means (e.g., Bolnick et al., 2011; Inouye, 2005), our results suggest that we should consider spatial variance in herbivory as well as mean herbivory.

## AUTHOR CONTRIBUTIONS

**Monica Paniagua Montoya:** Formal analysis (lead); writing – original draft (lead); writing – review and editing (equal). **Alexander J. Forde:** Conceptualization (equal); investigation (lead); writing – review and editing (equal). **Brian Inouye:** Formal analysis (supporting); writing – review and editing (equal). **Nora Underwood:** Conceptualization (equal); formal analysis (supporting); funding acquisition (lead); writing – review and editing (equal).

## CONFLICT OF INTEREST STATEMENT

The authors have no conflict of interests.

## Data Availability

Data and R code used for analysis are available at OSF project site (https://osf.io/cvbfw/).

## APPENDIX A

Our estimates of percent leaflet damage implicitly assume that any variation in leaflet size negligibly influences our results. To test this assumption, we used a simulation where we compared the qualitative differences between models that assumed no differences and size differences among leaflets (i.e., scale at which we estimated percent damage). We created two measures of absolute damage. The first measure assigned each leaflet a size of 82.5 cm^2^ (hereafter no variation area) which is the average leaflet size reported for various tomato cultivars (Kang & Sinha, 2010). For the second measure, we randomly assigned each leaflet an area from 5 – 160 cm^2^ (hereafter variation area), which brackets the average leaflet area reported for various tomato cultivars (Kang & Sinha, 2010). We used the estimated absolute damages for each leaflet (i.e., product of no variation or variation areas and percent damage divided by 100) to calculate measures of per capita damage and variation in damage (coefficient of variation of damage) for each plant. To determine the effect of leaflet variation on our results, we used linear mixed effects models (‘lme4’ package; Gaussian distribution) and a backward step‐wise approach to test combinations of additive and interactive effects among plant resistance, average herbivore density, and predation treatments. We compared the fits of models using Akaike's Information Criterion (AIC; ‘AICcmodavg’ package), and then a type II ANOVA for hypothesis testing (‘car’ package).

Our qualitative results do not differ when we assume variation in leaflet size (Figures A1 and A2, Table A1). The best‐fit models for per capita herbivore damage with and without leaflet variation both included the additive effects of average herbivore density, plant resistance, predation, and temporal block, and retained an interaction term between average herbivore density and plant resistance (Table A1). The best‐supported model for herbivore damage dispersion with and without leaflet variation both retained plant resistance, predator treatment, herbivore density, temporal block, and an interactive effect of plant resistance and herbivore density (Table A1).

**TABLE A1:** There are no qualitative differences between models that assume no variation in leaflet size and variation in leaflet size for per capita damage and variation in damage.

Predictor	Per capita damage				Variation in damage			
	No variation in leaflet size		Variation in leaflet size		No variation in leaflet size		Variation in leaflet size	
	*χ* ^2^‐value_df_	*p*‐value	*χ* ^2^‐value_df_	*p*‐value	*χ* ^2^‐value_df_	*p*‐value	*χ* ^2^‐value_df_	*p*‐value
Average density	**4.04**_**1**_	**.04**	**4.1**_**1**_	**.043**	**187.1**_**1**_	**<.0001**	**201.2**_**1**_	**<.0001**
Plant resistance	**107**_**2**_	**<.0001**	**105**_**2**_	**<.0001**	2.83_2_	.22	3.9_2_	.14
Predator	**8.0**_**1**_	**.005**	**6.98**_**1**_	**.008**	**4.48**_**1**_	**.03**	**4.61**_**1**_	**.032**
Plant resistance* Density	**12.3**_**2**_	**.002**	**13.1**_**2**_	**.001**	**54.1**_**2**_	**<.0001**	**46.3**_**2**_	**<.0001**
Block	**14.6**_**1**_	**.0001**	**13.5**_**1**_	**.0002**	0.19_1_	.67	0.003_1_	.96

*Note*: For each model, *X* ^*2*^ and degrees of freedom are shown. Terms with *p*‐values <.05 are in bold.

## APPENDIX B

Protease inhibitor assay.

We measured trypsin inhibitors, which are a type of serine PI that is known to induce resistance in tomato plants in response to herbivory (Jongsma et al., 1994). Generally, PIs are an important component of induced resistance to herbivores as they are rapidly expressed, systemic, and persistent, although they are not the only potential mechanism of resistance. To ensure that we had undamaged leaflets for assays, 3 days after experimental plants were sprayed with methyl jasmonate or water, we tied small cloth bags onto the most recently expanded leaves of two plants per enclosure; leaflets were harvested from within bags at the beginning (4 days after spraying) and end of the experiment (8 days after spraying). Previous studies indicate that plants respond differently to mechanical damage, such as clipping, compared to herbivore damage (e.g., Agrawal & Sherriffs, 2001; Baldwin, 1990). The clipping of a leaflet is not likely to induce resistance in a manner that later influenced the herbivores. Terminal leaflets on each leaf were chosen for collection unless they were unhealthy or mechanically damaged. We obtained a ground 0.100 g (+/− 0.010) tissue sample of each leaf. To extract proteinase inhibitors (PI), samples were vortexed with 800 uL of TRIS buffer (7.88 g TRIS–HCL in 900 mL ddH2O) for 30 s and centrifuged for 15 min at 11,000 rpm and 4°C. For each sample, we created the mixture A by combining 60 uL sample extract, 20 uL TRIS buffer, 50 uL substrate, and 20 uL enzyme. The mixture B included 60 uL sample extract, 40 uL TRIS buffer, and 50 uL substrate. The controls, C and D, included no sample extract, 80 or 100 uL TRIS buffer, 50 uL substrate, and 20 or 0 uL enzyme, respectively. Each mixture was vortexed and incubated for 20 min at 28°C. After adding 100 uL of 10% Trichloracetate, were centrifuged for 15 min. We used SoftMax Pro to read the absorbency values for each mixture. Percent of protease inhibition was measured as % Inhibition = [1‐(A‐B)/(C‐D)] x 100, where A and B represent absorbance values of each sample with and without the trypsin enzyme, respectively, and C and D represent absorbance values without the leaf sample and with or without enzyme, respectively. It is important to note that the equation assumes a similar rate of trypsin activity for the samples (A and B) and the samples without leaves (C and D), but there can be coenzymes and cofactors that can increase trypsin activity in the leaf samples resulting in an overall negative PI value.

## References

[ece370106-bib-0001] Agrawal, A. A. , & Karban, R. (1999). Why induced defenses may be favored over constitutive strategies in plants. In R. Tollrian , & C. D. Harvell (Eds.), The ecology and evolution of inducible defenses (pp. 45–61). Princeton University Press.

[ece370106-bib-0002] Agrawal, A. A. , & Sherriffs, M. F. (2001). Induced plant resistance and susceptibility to late‐season herbivores of wild radish. Annals of the Entomological Society of America, 94(1), 71–75. 10.1603/0013-8746(2001)094[0071:IPRAST]2.0.CO;2

[ece370106-bib-0003] Anderson, K. E. , Inouye, B. D. , & Underwood, N. (2015). Can inducible resistance in plants cause herbivore aggregations? Spatial patterns in an inducible plant/herbivore model. Ecology, 96(10), 2758–2770. 10.1890/14-1697.1 26649396

[ece370106-bib-0004] Aqueel, M. A. , & Leather, S. R. (2012). Nitrogen fertilizer affects the functional response and prey consumption of *Harmonia axyridis* (Coleoptera: Coccinellidae) feeding on cereal aphids. Annals of Applied Biology, 160(1), 6–15. 10.1111/j.1744-7348.2011.00514.x

[ece370106-bib-0005] Baldwin, I. T. (1990). Herbivory simulations in ecological research. Trends in Ecology & Evolution, 5(3), 91–93. 10.1016/0169-5347(90)90237-8 21232330

[ece370106-bib-0006] Bergelson, J. , Fowler, S. , & Hartley, S. (1986). The effects of foliage damage on casebearing moth larvae, *Coleophora serratella*, feeding on birch. Ecological Entomology, 11(3), 241–250. 10.1111/j.1365-2311.1986.tb00300.x

[ece370106-bib-0007] Bernays, E. A. (1997). Feeding by lepidopteran larvae is dangerous. Ecological Entomology, 22(1), 121–123. 10.1046/j.1365-2311.1997.00042.x

[ece370106-bib-0008] Berryman, A. A. , Stenseth, N. C. , & Isaev, A. S. (1987). Natural regulation of herbivorous forest insect populations. Oecologia, 71(2), 174–184. 10.1007/BF00377282 28312243

[ece370106-bib-0009] Bolnick, D. I. , Amarasekare, P. , Araújo, M. S. , Bürger, R. , Levine, J. M. , Novak, M. , Rudolf, V. H. W. , Schreiber, S. J. , Urban, M. C. , & Vasseur, D. A. (2011). Why intraspecific trait variation matters in community ecology. Trends in Ecology & Evolution, 26(4), 183–192. 10.1016/j.tree.2011.01.009 21367482 PMC3088364

[ece370106-bib-0010] Boughton, A. J. , Hoover, K. , & Felton, G. W. (2005). Methyl jasmonate application induces increased densities of glandular trichomes on tomato, *Lycopersicon esculentum* . Journal of Chemical Ecology, 31(9), 2211–2216. 10.1007/s10886-005-6228-7 16132222

[ece370106-bib-0011] Camara, M. D. (1997). Predator responses to sequestered plant toxins in buckeye caterpillars: Are tritrophic interactions locally variable? Journal of Chemical Ecology, 23(9), 2093–2106. 10.1023/B:JOEC.0000006431.34359.c2

[ece370106-bib-0012] Dalin, P. , Ågren, J. , Björkman, C. , Huttunen, P. , & Kärkkäinen, K. (2008). Leaf trichome formation and plant resistance to herbivory. In A. Schaller (Ed.), Induced plant resistance to herbivory (pp. 89–105). Springer. 10.1007/978-1-4020-8182-8_4

[ece370106-bib-0013] DeClercq, P. , & Degheele, D. (1994). Laboratory measurement of predation by *Podisus maculiventris* and *P. sagitta* (Hemiptera: Pentatomidae) on Beet Armyworm (Lepidoptera: Noctuidae). Journal of Economic Entomology, 87(1), 76–83. 10.1093/jee/87.1.76

[ece370106-bib-0014] Despland, E. (2019). Caterpillars cooperate to overcome plant glandular trichome defenses. Frontiers in Ecology and Evolution, 7, 232. 10.3389/fevo.2019.00232

[ece370106-bib-0015] Dicke, M. (2015). Herbivore‐induced plant volatiles as a rich source of information for arthropod predators: Fundamental and applied aspects. Journal of the Indian Institute of Science, 95(1), 35–42.

[ece370106-bib-0016] Edwards, P. J. , & Wratten, S. D. (1983). Wound induced defenses in plants and their consequences for patterns of insect grazing. Oecologia, 59(1), 88–93. 10.1007/BF00388079 25024154

[ece370106-bib-0017] Ghent, A. W. (1960). A study of the group‐feeding behaviour of larvae of the Jack pine sawfly, *Neodiprion Pratti Banksianae* Roh. Behaviour, 16(1–2), 110–147. 10.1163/156853960X00070

[ece370106-bib-0018] Hairston, N. G. , Smith, F. E. , & Slobodkin, L. B. (1960). Community structure, population control, and competition. The American Naturalist, 94(879), 421–425. 10.1086/282146

[ece370106-bib-0019] Hermann, S. L. , & Thaler, J. S. (2014). Prey perception of predation risk: Volatile chemical cues mediate non‐consumptive effects of a predator on a herbivorous insect. Oecologia, 176(3), 669–676. 10.1007/s00442-014-3069-5 25234373

[ece370106-bib-0020] Holling, C. S. (1961). Principles of insect predation. Annual Review of Entomology, 6(1), 163–182. 10.1146/annurev.en.06.010161.001115

[ece370106-bib-0021] Inouye, B. D. (2005). The importance of the variance around the mean effect size of ecological processes: Comment. Ecology, 86(1), 262–265. 10.1890/03-3180

[ece370106-bib-0022] Inouye, B. D. , & Johnson, D. M. (2005). Larval aggregation affects feeding rate in *Chlosyne poecile* (Lepidoptera: Nymphalidae). Florida Entomologist, 88(3), 247–252. 10.1653/0015-4040(2005)088[0247:LAAFRI]2.0.CO;2

[ece370106-bib-0023] Johnson, M. T. J. (2008). Bottom‐up effects on plant genotype on aphids, ants, and predators. Ecology, 89(1), 145–154. 10.1890/07-0395.1 18376556

[ece370106-bib-0069] Jongsma, M. A. , Bakker, P. L. , Visser, B. , & Stiekema, W. J. (1994). Trypsin inhibitor activity in mature tobacco and tomato plants is mainly induced locally in response to insect attack, wounding and virus infection. Planta, 195(1). 10.1007/bf00206288

[ece370106-bib-0024] Kang, J. , & Sinha, N. R. (2010). Leaflet initiation is temporally and spatially separated in simple and complex tomato (*Solanum lycopersicum*) leaf mutants: A developmental analysis. Botany, 88(8), 710–724. 10.1139/B10-051

[ece370106-bib-0025] Kaplan, I. , McArt, S. H. , & Thaler, J. S. (2014). Plant defenses and predation risk differentially shape patterns of consumption, growth, and digestive efficiency in a guild of leaf‐chewing insects. PLoS One, 9(4), e93714. 10.1371/journal.pone.0093714 24718036 PMC3981721

[ece370106-bib-0026] Kaplan, I. , & Thaler, J. S. (2010). Plant resistance attenuates the consumptive and non‐consumptive impacts of predators on prey. Oikos, 119(7), 1105–1113. 10.1111/j.1600-0706.2009.18311.x

[ece370106-bib-0027] Karban, R. (1987). Effects of clonal variation of the host plant, interspecific competition, and climate on the population size of a folivorous thrips. Oecologia, 74(2), 298–303. 10.1007/BF00379373 28312004

[ece370106-bib-0028] Karban, R. , & Agrawal, A. A. (2002). Herbivore offense. Annual Review of Ecology and Systematics, 33(1), 641–664. 10.1146/annurev.ecolsys.33.010802.150443

[ece370106-bib-0029] Karban, R. , & English‐Loeb, G. M. (1988). Effects of herbivory and plant conditioning on the population dynamics of spider mites. Experimental & Applied Acarology, 4(3), 225–246. 10.1007/BF01196188

[ece370106-bib-0030] Karban, R. , & Myers, J. H. (1989). Induced plant responses to herbivory. Annual Review of Ecology and Systematics, 20, 331–348.

[ece370106-bib-0031] Kersch‐Becker, M. F. , Kessler, A. , & Thaler, J. S. (2017). Plant defences limit herbivore population growth by changing predator–prey interactions. Proceedings of the Royal Society B: Biological Sciences, 284(1862), 20171120. 10.1098/rspb.2017.1120 PMC5597831 28878062

[ece370106-bib-0032] Kersch‐Becker, M. F. , & Thaler, J. S. (2015). Plant resistance reduces the strength of consumptive and non‐consumptive effects of predators on aphids. Journal of Animal Ecology, 84(5), 1222–1232. 10.1111/1365-2656.12371 25788108

[ece370106-bib-0033] Kersch‐Becker, M. F. , & Thaler, J. S. (2019). Constitutive and herbivore‐induced plant defences regulate herbivore population processes. Journal of Animal Ecology, 88(7), 1079–1088. 10.1111/1365-2656.12993 30968954

[ece370106-bib-0034] Kolodny‐Hirsch, D. M. , Warkentin, D. L. , Alvarado‐Rodriguez, B. , & Kirkland, R. (1993). *Spodoptera exigua* nuclear polyhedrosis virus as a candidate viral insecticide for the beet armyworm (Lepidoptera: Noctuidae). Journal of Economic Entomology, 86(2), 314–321. 10.1093/jee/86.2.314

[ece370106-bib-0035] Li, C. , Williams, M. M. , Loh, Y.‐T. , Lee, G. I. , & Howe, G. A. (2002). Resistance of cultivated tomato to cell content‐feeding herbivores is regulated by the octadecanoid‐signaling pathway. Plant Physiology, 130(1), 494–503. 10.1104/pp.005314 12226528 PMC166581

[ece370106-bib-0036] Li, L. , Li, C. , & Howe, G. A. (2001). Genetic analysis of wound signaling in tomato. Evidence for a dual role of jasmonic acid in defense and female fertility. Plant Physiology, 127(4), 1414–1417. 10.1104/pp.010705 11743083 PMC1540172

[ece370106-bib-0037] Marquis, R. J. (1992). A bite is a bite is a bite? Constraints on response to folivory in *piper Arieianum* (Piperaceae). Ecology, 73(1), 143–152. 10.2307/1938727

[ece370106-bib-0038] Mauricio, R. , Bowers, M. D. , & Bazzaz, F. A. (1993). Pattern of leaf damage affects fitness of the annual plant *Raphanus Sativus* (Brassicaceae). Ecology, 74(7), 2066–2071. 10.2307/1940852

[ece370106-bib-0039] McNutt, D. W. , Samuelson, K. , & Underwood, N. (2017). Pathways for plant‐mediated negative feedback to insect herbivores: Accounting for non‐linear effects of larval density on plant quality and quantity. Entomologia Experimentalis et Applicata, 162(1), 93–104. 10.1111/eea.12515

[ece370106-bib-0040] Morris, W. F. , Traw, M. B. , & Bergelson, J. (2006). On testing for a tradeoff between constitutive and induced resistance. Oikos, 112(1), 102–110. 10.1111/j.0030-1299.2006.14253.x

[ece370106-bib-0041] Mutz, J. , Underwood, N. , & Inouye, B. D. (2020). Integrating top‐down and bottom‐up effects of local density across scales and a complex life cycle. Ecology, 101(10), e03118. 10.1002/ecy.3118 32531072

[ece370106-bib-0042] Orians, C. M. , Pomerleau, J. , & Ricco, R. (2000). Vascular architecture generates fine scale variation in systemic induction of proteinase inhibitors in tomato. Journal of Chemical Ecology, 26(2), 471–485.

[ece370106-bib-0043] Orrock, J. , Connolly, B. , & Kitchen, A. (2017). Induced defenses in plants reduce herbivory by increasing cannibalism. Nature Ecology & Evolution, 1(8), 1205–1207. 10.1038/s41559-017-0231-6 29046571

[ece370106-bib-0044] Preisser, E. L. , Bolnick, D. I. , & Benard, M. F. (2005). Scared to death? Effects of intimidation and consumption in predator‐prey interactions. Ecology, 86(2), 501–509. 10.1890/04-0719

[ece370106-bib-0045] Price, P. W. , Bouton, C. E. , Gross, P. , McPheron, B. A. , Thompson, J. N. , & Weis, A. E. (1980). Interactions among three trophic levels: Influence of plants on interactions between insect herbivores and natural enemies. Annual Review of Ecology and Systematics, 11(1), 41–65. 10.1146/annurev.es.11.110180.000353

[ece370106-bib-0046] R Core Team . (2022). R: A language and environment for statistical computing. R Foundation for Statistical Computing. https://www.R‐project.org/

[ece370106-bib-0048] Riddick, E. W. , & Simmons, A. M. (2014). Do plant trichomes cause more harm than good to predatory insects?: Plant trichomes and predators. Pest Management Science, 70(11), 1655–1665. 10.1002/ps.3772 24585676

[ece370106-bib-0049] Rodriguez‐Saona, C. , & Thaler, J. S. (2005). The jasmonate pathway alters herbivore feeding behaviour: Consequences for plant defences. Entomologia Experimentalis et Applicata, 115(1), 125–134. 10.1111/j.1570-7458.2005.00277.x

[ece370106-bib-0050] Rotem, K. A. , & Agrawal, A. A. (2003). Density dependent population growth of the two‐spotted spider mite, *Tetranychus urticae*, on the host plant *Leonurus cardiaca* . Oikos, 103(3), 559–565. 10.1034/j.1600-0706.2003.12531.x

[ece370106-bib-0051] Schmitz, O. J. , Beckerman, A. P. , & O'Brien, K. M. (1997). Behaviorally mediated trophic cascades: Effects of predation risk on food web interactions. Ecology, 78(5), 1388–1399. 10.1890/0012-9658(1997)078[1388:BMTCEO]2.0.CO;2

[ece370106-bib-0052] Sheriff, M. J. , Peacor, S. D. , Hawlena, D. , & Thaker, M. (2020). Non‐consumptive predator effects on prey population size: A dearth of evidence. Journal of Animal Ecology, 89(6), 1302–1316. 10.1111/1365-2656.13213 32215909

[ece370106-bib-0053] Smits, P. H. , Velden, M. C. , Vrie, M. , & Vlak, J. M. (1987). Feeding and dispersion of *Spodoptera exigua* larvae and its relevance for control with a nuclear polyhedrosis virus. Entomologia Experimentalis et Applicata, 43(1), 67–72. 10.1111/j.1570-7458.1987.tb02204.x

[ece370106-bib-0054] Stiling, P. (1988). Density‐dependent processes and key factors in insect populations. The Journal of Animal Ecology, 57(2), 581. 10.2307/4926

[ece370106-bib-0055] Stout, M. J. , & Duffey, S. S. (1996). Characterization of induced resistance in tomato plants. Entomologia Experimentalis et Applicata, 79(3), 273–283. 10.1111/j.1570-7458.1996.tb00835.x

[ece370106-bib-0056] Stout, M. J. , Workman, K. V. , Workman, J. S. , & Duffey, S. S. (1996). Temporal and ontogenetic aspects of protein induction in foliage of the tomato, *Lycopersicon esculentum* . Biochemical Systematics and Ecology, 24, 611–625.

[ece370106-bib-0057] Thaler, J. S. , Contreras, H. , & Davidowitz, G. (2014). Effects of predation risk and plant resistance on *Manduca sexta* caterpillar feeding behaviour and physiology: Predation affects prey behaviour and physiology. Ecological Entomology, 39(2), 210–216. 10.1111/een.12086

[ece370106-bib-0058] Thaler, J. S. , & Griffin, C. A. M. (2008). Relative importance of consumptive and non‐consumptive effects of predators on prey and plant damage: The influence of herbivore ontogeny. Entomologia Experimentalis et Applicata, 128(1), 34–40. 10.1111/j.1570-7458.2008.00737.x

[ece370106-bib-0059] Thaler, J. S. , Stout, M. J. , Karban, R. , & Duffey, S. S. (1996). Exogenous jasmonates simulate insect wounding in tomato plants (*Lycopersicon esculentum*) in the laboratory and field. Journal of Chemical Ecology, 22(10), 1767–1781. 10.1007/BF02028503 24227107

[ece370106-bib-0060] Thaler, J. S. , Stout, M. J. , Karban, R. , & Duffey, S. S. (2001). Jasmonate‐mediated induced plant resistance affects a community of herbivores: Induced resistance affects the herbivore community. Ecological Entomology, 26(3), 312–324. 10.1046/j.1365-2311.2001.00324.x

[ece370106-bib-0061] Turchin, P. (2003). Complex population dynamics: A theoretical/empirical synthesis. Princeton University Press. 10.1515/9781400847280

[ece370106-bib-0062] Turlings, T. C. , Tumlinson, J. H. , & Lewis, W. J. (1990). Exploitation of herbivore‐induced plant odors by host‐seeking parasitic wasps. Science, 250(4985), 1251–1253. 10.1126/science.250.4985.1251 17829213

[ece370106-bib-0063] Uesugi, A. (2015). The slow‐growth high‐mortality hypothesis: Direct experimental support in a leaf mining fly. Ecological Entomology, 40(3), 221–228. 10.1111/een.12177

[ece370106-bib-0064] Underwood, N. (2000). Density dependence in induced plant resistance to herbivore damage: Threshold, strength and genetic variation. Oikos, 89(2), 295–300. 10.1034/j.1600-0706.2000.890210.x

[ece370106-bib-0065] Underwood, N. (2010). Density dependence in insect performance within individual plants: Induced resistance to *Spodoptera exigua* in tomato. Oikos, 119(12), 1993–1999. 10.1111/j.1600-0706.2010.18578.x

[ece370106-bib-0066] Underwood, N. , Anderson, K. , & Inouye, B. D. (2005). Induced vs. constitutive resistance and the spatial distribution of insect herbivores among plants. Ecology, 86(3), 594–602. 10.1890/03-0290

[ece370106-bib-0067] Vidal, M. C. , & Murphy, S. M. (2018). Bottom‐up vs. top‐down effects on terrestrial insect herbivores: A meta‐analysis. Ecology Letters, 21(1), 138–150. 10.1111/ele.12874 29098754

[ece370106-bib-0068] Zheng, X.‐L. , Cong, X.‐P. , Wang, X.‐P. , & Lei, C.‐L. (2011). A review of geographic distribution, overwintering and migration in *Spodoptera exigua* Hübner (Lepidoptera: Noctuidae). Journal of the Entomological Research Society, 13(3), 39–48.

